# Jaw Laterality and Related Handedness in the Hunting Behavior of a Scale-Eating Characin, *Exodon paradoxus*

**DOI:** 10.1371/journal.pone.0029349

**Published:** 2011-12-28

**Authors:** Hiroki Hata, Masaki Yasugi, Michio Hori

**Affiliations:** 1 Graduate School of Science and Technology, Ehime University, Matsuyama, Ehime, Japan; 2 Graduate School of Science, Kyoto University, Kyoto, Japan; Institute of Marine Research, Norway

## Abstract

**Background:**

Asymmetry in animal bodies and behavior has evolved several times, but our knowledge of their linkage is limited. Tanganyikan scale-eating cichlids have well-known antisymmetry in their bodies and behavior; individuals open their mouths leftward (righty) or rightward (lefty), and righties always attack the right flank of the prey, whereas lefties attack the left. This study analyzed the morphological asymmetry in a scale-eating characiform, *Exodon paradoxus*, and its behavioral handedness.

**Methodology/Principal Findings:**

Each eight *E. paradoxus* was observed for 1-h with a prey goldfish in an aquarium to detect the behavioral handedness. Following the experiment, the lateral differences in the mandibles and head-inclination of these eight and ten additional specimens were analyzed. Both measurements on the morphology showed a bimodal distribution, and the laterality identified by these two methods was always consistent within a given individual, indicating that the characin has morphological antisymmetry. Furthermore, this laterality significantly corresponded to behavioral handedness; that is, lefties more often rasped scales from the right flank of the prey and vice versa. However, the correlation between laterality and handedness is the opposite of that in the cichlids. This is due to differences in the feeding apparatus and technique. The characin has cuspids pointing forward on the external side of the premaxilla, and it thrusts its dominant body side outward from its body axis on the flank of the prey to tear off scales. By contrast, the cichlids draw their dominant body side inward toward the axis or rotate it to scrape or wrench off scales with the teeth lined in the opened mouth.

**Conclusions/Significance:**

This study demonstrated that the antisymmetry in external morphology and the corresponding behavioral handedness have evolved in two lineages of scale-eating fishes independently, and these fishes adopt different utilization of their body asymmetry to tear off scales.

## Introduction

Antisymmetry is a phenomenon in which a population of a species are consist of right-sided and left-sided, or dextral and sinistral, forms with even frequencies [1]. Lateral asymmetry in animal can be classified by the shape of histogram into 3 categories; fluctuating asymmetry (FA) with unimodal and symmetric distribution, directional asymmetry (DA) with unimodal distribution shifted from symmetry, and anti-symmetry (AS) with bimodal distribution. Antisymmetry in external morphology is widely known in nature and has evolved several times in more than 450 species from 67 families in eight phyla, including lobsters, crossbill birds, and scale-eating cichlids [1]. The bimodal laterality in the jaw morphology and head inclination of a scale-eating cichlid, *Perissodus microlepis* [2], [3], an herbivorous Tanganyikan cichlid, *Neolamprologus moorii* [3], a Japanese riverine goby, *Rhinogobius flumineus* [4], the Japanese medaka *Oryzias latipes* [5], the zebrafish *Danio rerio* [6] and a Tanganyikan cichlid, *Julidochromis ornatus* [5], are suggested to be genetically determined.

Behavioral asymmetry, in which every individual has either left- or right-biased behavior, is revealed in the detour behavior and eye use of fishes [7], mouth use of fishes [8], [9], and paw/hand use of toads [10], mice [11], cats [12], and chimpanzees [13]. Recent studies have revealed that some behavioral handedness is highly correlated with antisymmetry in external morphology. For example, handedness in foraging behavior is correlated with laterality in mouth opening in scale-eating cichlids [2], [14], the shrimp-eating cichlid, *Neolamprologus fasciatus* [9], and large-mouth bass, *Micropterus salmoides* [8]. Furthermore, handedness during lateral display in male–male competition is correlated with head inclination laterality in the Siamese fighting fish, *Betta splendens* [15].

The scale-eating cichlid is one of the best-known examples of both morphological and behavioral antisymmetry. Scale eating is highly specialized foraging in which the prey is usually bigger than the predator, so the scale eater needs to adapt to the high motility and possible counterattack by its prey [16]. Furthermore, since lepidophagy does not kill the prey, prey fishes avoid predators by learning, as well as by intrinsic behavior [2], [17]. Therefore, scale-eating fish have both specialized feeding apparatus and behavior, and they have evolved descaling teeth [18], [19], multiple approaching strategies [20], [21], relevant concealing colorations [22], and even aggressive mimicry [23], [24]. Some fishes adopt a remarkable adaptation for scale-eating, that is, laterality. In all seven species of scale-eating cichlid in Lake Tanganyika, every individual opens its mouth either rightward (lefty) or leftward (righty) as a result of the asymmetric position of the joints of the mandibles and the suspensorium [18], [25], [26]. Note that the definition of laterality used in recent studies differs from the definition used in the early papers [2], [25]. These earlier papers defined individuals with the mouth opening to the right as “right-handed”, or “dextral”. The terminology used in the present study, “lefty”, reflects the fact that the left mandible of such “right-handed” fish is larger than their right mandibles [3], [9], [27] and that their left eye is the dominant eye [9], [15]. The direction in this laterality is determined genetically by a one-locus two-allele Mendelian system, with the lefty dominant over the righty [2], [3]. Furthermore, in the cichlids *Perissodus microlepis* and *P. straeleni*, under natural conditions, righty individuals always attack the right flank of their prey, and lefties attack the left flank [2], [3]. This biased attack causes the prey fishes being vigilant to one side of their body that is attacked more frequently, and therefore numerically dominant morph (either lefty or righty) of *P. microlepis* decreases their predation success [2]. This is a case of the negative frequency-dependent selection, and the frequency of lefty and righty morphs oscillates around unity [2]. An experimental study of the laterality of *P. microlepis* demonstrated some morphological and behavioral plasticity in an artificial environment [28].

Scale-eating habits are known in five freshwater and eight marine fish families (Table S1), and have evolved at least 12 times independently [16], [29]. In addition to Tanganyikan cichlids, only the scale-eating triacanthodid *Macrorhamphosodes uradoi* is recognized as having either a leftward- or rightward-twisted mouth [29]. Morphological investigation reveals that the scales in the stomachs of *M. uradoi* are stolen from the caudal fin and the base of prey fishes, and therefore, *M. uradoi* is supposed to attack their prey from behind. In that attacking behavior asymmetric mouth may have a function, but it remains unknown how it actually works in deep water.

Another diverse, well-studied group exhibiting lepidophagy is the freshwater characins of South America. Six genera, including *Exodon*, are scale eaters [16], [30]. Fish scales fill 88% of the stomach contents in *E. paradoxus* collected in the wild [31]. Characiformes is a relatively older teleostean group that lacks the apparatus for upper jaw premaxilla protrusion that the newer scale-eating fishes have achieved [32]. Instead, scale-eating characins have enlarged cuspidate teeth that point forward on the labial sides of the jaws [33]. *E. paradoxus* has a pair of cuspidate teeth on the premaxilla [34] and rushes at the flank of its prey with its mouth either open or closed [16].

This study examined the mechanism by which characin scale eaters remove scales compared with other scale-eating fishes and whether the antisymmetry in mouth morphology and related behavioral handedness are ubiquitous in scale-eating fishes. Therefore, we observed the feeding behavior of a wild-caught scale-eating characin, *E. paradoxus*, and examined its morphological laterality, its behavioral handedness in hunting.

## Materials and Methods

### Behavioral observations

This study was performed in accordance with the Regulation on Animal Experimentation at Kyoto University. Approval is not needed for fishes under Japanese law, Act on Welfare and Management of Animals. Eight wild-caught adult *Exodon paradoxus* [52.2±5.7-mm standard length (SL), average ± standard deviation (SD)], imported from Colombia, were obtained from a commercial vendor. *E. paradoxus* feed on scales with 3–5 mm in diameter [30], therefore, live goldfish, *Carassius auratus auratus* (63.2±5.5-mm SL), were used as prey that have the similar scale size. After starvation for 24 hours, each *E. paradox* individual was transferred to a 58-L aquarium with one prey individual, with the two separated by a partition. After 15 min of acclimation, the partition was removed, and foraging events were recorded for 1-h using a digital video camera (Panasonic SDR-H80). The attacked flank of the prey, which direction (anterior or posterior of the prey) *E. paradoxus* turned its jaws when tearing off scales, and the moment when the feeding behavior occurred during the 1-hour observation were recorded. Each *E. paradoxus* and goldfish pair was observed once.

### Measurement of morphological asymmetries

Following the observations, the fish were sacrificed using an overdose of 2-phenoxyethanol and fixed in 10% formalin solution. Ten additional *E. paradoxus* (49.9±4.1-mm SL) were obtained from the same vendor and fixed in the same way.

**Figure 1:**
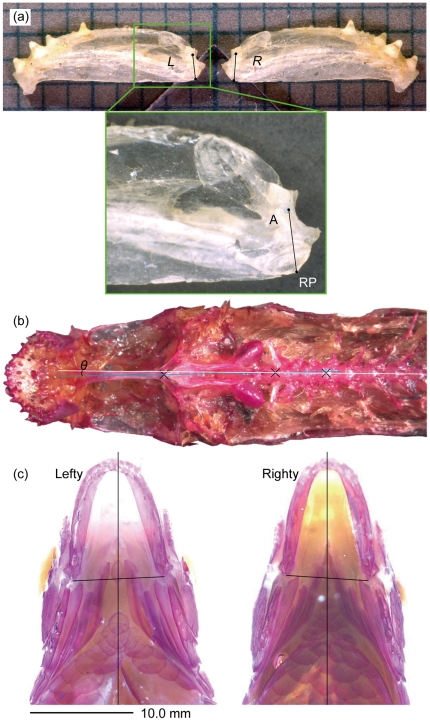
Measurement points of laterality in *Exodon paradoxus* for the mandible height and head inclination. Photographs of (a) the left and right mandibles and the height measurements (*L* and *R*) between the socket bottom and ventral corner of the retroarticular, (b) the head and vertebrae in the ventral views and their angle (*θ*), and (c) transparent specimens of lefty and righty individuals in the ventral view. Note that in (c), the lines between the right and left ventral corners of the retroarticular do not cross perpendicularly to the vertical lines that indicate the midlines of the bodies, but rather slant to the left or right in the lefty and righty, respectively.

To evaluate morphological laterality, two measurements were examined (Fig. 1): (1) the difference between the heights of the right and left posterior end of the mandible (HMPE), measured as the distance between the socket bottom of the suspensorial articulation facet of the anguloarticular and the most inferior point of the retroarticular process [3], and (2) head inclination, *i*.*e*., the angle (*θ*) between a line from the center of the parasphenoid to the center of the basioccipital and another line from the center of the basioccipital to the center of the third spinal segment in the ventral view [15], [27]. The right-left difference of the HMPE means the following: the HMPE acts as a line between the effort point (where the ligament is attached) and the fulcrum point (the articulation part of the mandible) of the lever [35], [36]. Thus, the difference between right and left HMPE itself may produce differential opening force and speed between right and left mandible, which necessarily cause the twisted mouth-opening. The morphological implication of the head-vertebrae angle is understood as a differential development of the right and left sides, the more developed side should be convex. Consequently the dominant side of the head faces front. Specimens were dissected to extract the mandibles and expose data points, which were marked and kept horizontal using a level-scope and then measured using a digital microscope (VHX-100, Keyence). Three measurements were made for each individual to reduce the observation error. The measurement error (ME) for our measurements were estimated as the proportion of within-individual variation to total variation, i.e., ME = *MS*_within_/(*S^2^_A_*+*MS*_wititin_)×100 (%), where *MS* is the mean square value and *S^2^_A_* is the added variance component. *S^2^_A_* was estimated as (*MS*_among_−*MS*_within_)/*n* from a one-way ANOVA with individuals as the fixed factor, where *n* is the number of repeated measures per individual, which in this study is 3 [37], [38]. The measurement errors were small (for the height of the posterior mandible ends, one-way ANOVA: *F_35,72_* = 8110, *p*<0.001, ME = 0.04%; for head inclination, one-way ANOVA: *F_17,36_* = 26701, *p*<0.001, ME = 0.01%). Therefore, mean values were used for the analysis.

An index of asymmetry (IAS) was calculated as follows: [2×(*R*−*L*)/(*R*+*L*)]×100, where *R* and *L* are the heights of the right and left mandibles, respectively [3]. Individuals with *R*>*L* were defined as righty because their right sides dominated over the left, and the IAS was assigned a positive value. By contrast, individuals with *R*<*L* were lefty, and IAS was negative. For the angle (*θ*), righty was defined as an individual whose right side of the head faced front, and the neurocranium bent rightward in the ventral view (Fig. 1b), and *θ* was given a positive value. By contrast, lefty, in which the left side of the head faced front and the neurocranium bent leftward in the ventral view, had a negative *θ*.

### Statistical analysis

The distributions of IAS and *θ* were fitted to the following three models: FA, with a normal distribution, mean 0, and SD of data; DA, with a normal distribution, mean≠0, and SD of data; and AS, with two normal distributions ± mean and SD calculated by the maximum likelihood estimation. The Akaike information criterion (AIC) was calculated for each model. The correlation between morphological and behavioral laterality was tested using a generalized linear mixed model (GLMM) with Poisson distribution using the attacked flank as a dependent variable (“0” was provided for the right flank, “1” was provided for the left flank), the morphological laterality (IAS or *θ*) and the moment of attacks as fixed factors, and the individual as a random factor. The frequencies of tear-off directions (anterior or posterior of the prey) toward which *E. paradoxus* turned its jaws when it attacked were compared using the Wilcoxon signed-rank test. All model fitting and analyses were performed using R2.11.0 [39].

## Results

### Scale-eating techniques of *Exodon paradoxus*

The scale-eating behavior was observed 5–96 (average 34.4±29.1 SD) times/hour in eight *E. paradoxus* individuals. *E. paradoxus* dashed forward to the flank of the prey from the side, pressed its snout against the flank, and then turned to the posterior of the prey (Video S1). This tear off direction was seen in 73.6±16.3% (average ± SD) attacks in eight individuals, and the opposite direction, *i*.*e*., to the anterior direction of the prey, in 19.1±14.8%. In the remaining 7.3±13.9% of attacks, *E. paradoxus* did not turn and instead hit the prey straight on. *E. paradoxus* tore off scales in a posterior direction of the prey significantly more often than the anterior direction (Wilcoxon signed-rank test, *Z* = 2.52, *p*<0.05). Such strikes caused scales to detach from the flank of the prey and float in the water column or settle to the substrate; the attacking *E. paradoxus* then fed on the scales.

### Laterality in morphology

**Figure 2:**
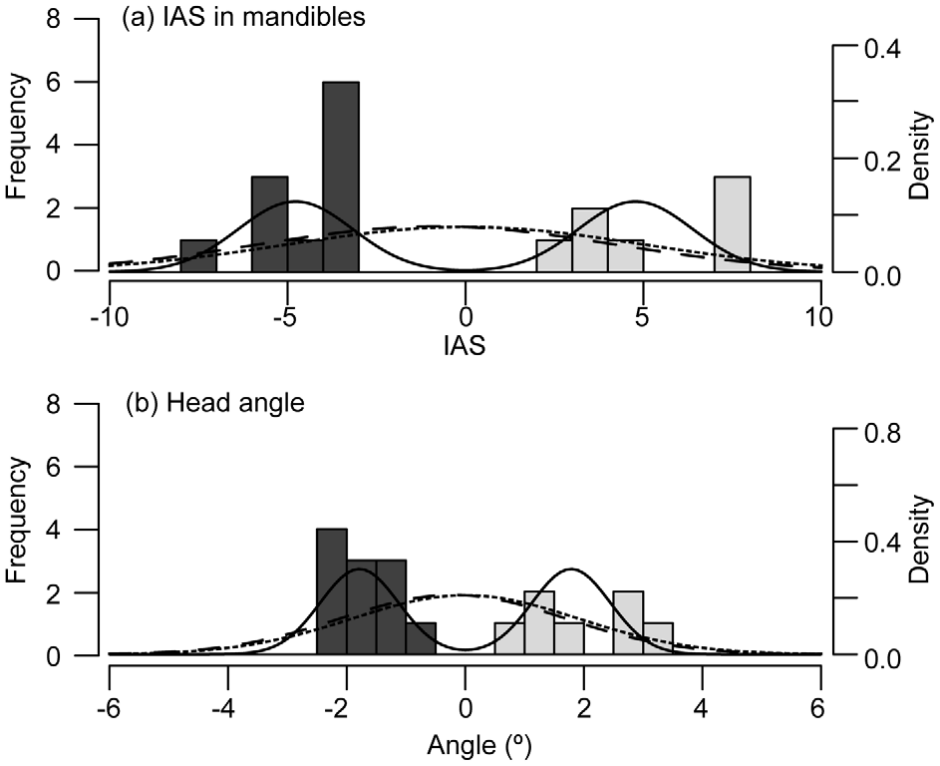
Frequency distributions of (a) the IAS of the mandibles and (b) head angle of *Exodon paradoxus*. The lines quantified by the second *y*-axis show the probability curves derived from the three models: the FA-model (dotted line), DA-model (broken line), and AS-model (solid line). Dark-grey bars indicate mandible lefties, and light-grey bars indicate righties.

Individual differences in the height of the mandibles between left and right sides and in the direction of inclination of the head were detected (Table 1, Fig. 2). In all individuals, the dominant sides were concordant between the mandibles and head angle. The model selection using the AIC showed that the distributions of the IAS of the mandibles and head angles were best fitted to the AS model (Table 2).

**Table 1:** Jaw laterality and frequency of attacks on the left flank or the right flank of the prey goldfish during the one-hour observation of eight *E. paradoxus.*

*Exodon paradoxus*		Frequency in which *E. paradoxus* attacked on prey goldfish/1 hour		
Individual code	Jaw laterality	Left flank	Right flank	Total number of attacks
1	lefty	20	24	44
2	lefty	15	26	41
5	lefty	10	25	35
7	lefty	5	7	12
10	lefty	2	3	5
4	righty	17	16	33
6	righty	6	3	9
9	righty	50	46	96

**Table 2:** AIC values for the three models to discriminate the type of asymmetry based on the two measures.

			AIC		
	Mean	SD	FA model	DA model	AS model
IAS	4.78	1.66	111.33	112.85	**97.21**
Angle	1.79	0.68	76.26	78.04	**64.97**

Bold indicates the minimum value among the AICs for the three models.

### Handedness in scale-eating behavior

**Figure 3:**
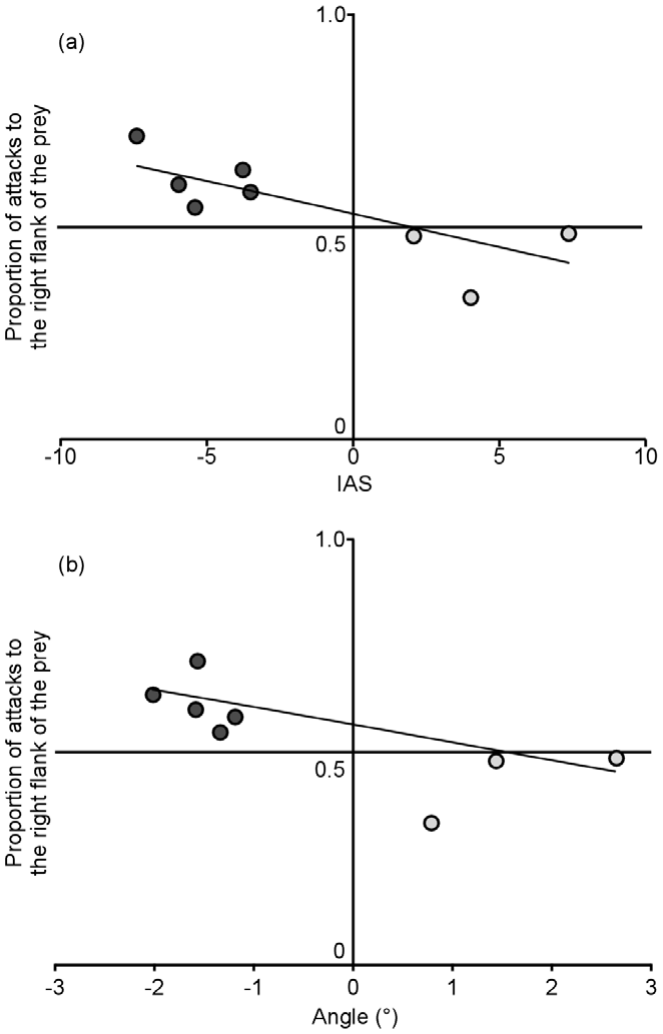
The correlation between morphological laterality and behavioral handedness in *Exodon paradoxus*. Scatter plots of the proportion of right-flank attacks by each individual and the IAS of the mandible (a) or head angle (b). Dark-grey points indicate lefties, and light-grey plots indicate righties. The solid lines are the fitted lines for the GLMMs.

The flank of the prey that *E. paradoxus* attacked was correlated significantly with the morphological laterality, both for the IAS of the mandible and the head angle (Fig. 3, both GLMMs, *p*<0.05, Tables 3,4). Righty individuals attacked left flanks more frequently than right flanks, and vice versa. This tendency was consistent during the 1-h observations (both GLMMs, *NS*).

**Table 3:** Results of GLMMs with morphological laterality, IAS and the timings when the attacks occurred as two fixed factors, the individual as a random factor, and which flank of the prey was attacked as a dependent variable.

	Estimate	Std. Error	*z* value	*p*
Intercept	0.09	0.22	0.40	0.69
IAS	−0.06	0.03	−2.44	0.01
Timings of attacks	0.00	0.00	0.21	0.84

**Table 4:** Results of GLMMs with morphological laterality, *θ*, and the timings when the attacks occurred as two fixed factors, the individual as a random factor, and which flank of the prey was attacked as a dependent variable.

	Estimate	Std. Error	*z* value	*p*
Intercept	0.10	0.22	0.48	0.63
*θ*	−0.18	0.07	−2.41	0.02
Timings of attacks	0.00	0.00	0.47	0.64

## Discussion

**Figure 4:**
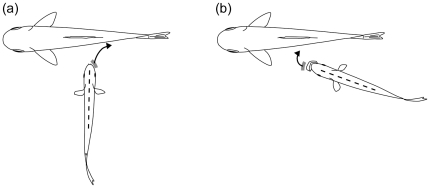
Behavioral handedness in scale-eating of (a) righty *Exodon paradoxus* and (b) lefty *Perissodus straeleni*. Arrows indicate the direction in which the scale-eaters hit their prey with its dominant jaw (indicated with gray bar) to scrape scales. Dashed line indicates the body axis of the scale-eaters. Note that the upper jaw of *P. straeleni* is protruded forward, but the upper jaw of *E. paradoxus* is fixed.

The scale-eating characin *Exodon paradoxus* exhibits morphological antisymmetry in its mandibles and concordant head inclination. Consequently, every individual has either a left- or right-dominant side of the mandible in parallel with head inclination, which places the dominant side forward. Furthermore, the morphological laterality correlates significantly with behavioral handedness, *i*.*e*., lefty individuals have a tendency to tear scales from the right flank of the prey, and vice versa. However, the correlation between laterality and handedness is the opposite of that in the scale-eating cichlids *Perissodus microlepis* and *P. straeleni* in which lefty attacks left flank [2], [14]. This discrepancy seems to be caused by the variation in feeding apparatus and technique; that is, characin scale eaters thrust the dominant side of the mouth outward from the body axis on the flank of the prey to tear off scales, whereas the cichlid scale eaters draw the dominant side inward to the axis or rotate it instead (Fig. 4). *E. paradoxus* rushes at the flank of the prey perpendicularly with its mouth open or closed [16], presses the external teeth pointed forward against the scales, and then turns to the posterior of the prey to tear off scales. The maxilla is fixed to the neurocranium in characins and provides effective force transfer at the hit, mediated by the external teeth [40]. Conversely, scale-eating cichlids dash at their prey from behind with the mouth opened wide because of their protruding jaws. They tear scales off in two ways [19]: they move their mouths laterally along the flank of the prey to scrape scales off using the edges of recurved laminar teeth arrayed in the jaws (*e*.*g*., *P. straeleni*), or they press their mouths against the flank of the prey and then rotate to wrench scales off with broad-based truncated teeth arrayed in their jaws (*e*.*g*., *P. microlepis*). The latter technique has evolved twice from the former [41]. In this way, characins and cichlids have independently acquired specialized antisymmetric apparatuses for scale eating, as well as the opposite correspondence between jaw laterality and behavioral handedness, with apparent phylogenetic constraints.

In *P. microlepis* and *P. straeleni*, righty individuals always attack the right flank of the prey and lefties attack the left flanks in nature [2], [3], and even in an aquarium [42]. In another aquarium, however, *P. microlepis* shows only weak correspondence between the morphological laterality and behavioral handedness possibly because the habituation of the fish to the artificial environment and prey [28]. Therefore, field observation on feeding behavior of *E. paradoxus* is needed to confirm the actual intensity of the morphology-behavior correspondence.

The conspicuous antisymmetry in external morphology and behavioral handedness and their correspondence have evolved independently in the two lineages of scale-eating fishes. Scale-eating has evolved in at least twelve families of fishes (Table S1), and only *E. paradoxus* of Characidae, *P. microlepis* and *P. straeleni* of Cichlidae are now known to have the both morphological laterality and the related handedness in scale-eating behavior [2], [3]. The other five Tanganyikan *Perissodus* species [1], [25], [26] and *Macrorhamphosodes uradoi* of Triacanthoidae [29] have the same morphological laterality, and therefore it is highly possible that they have the behavioral handedness corresponding to their morphological laterality.

In East Africa, one cichlid species, *Haplochromis welcommei*, in Lake Victoria [43], four cichlids, *Genyochromis mento, Corematodus toeniatus*, *C. shiranus,* and *Docimodus evelynae*, in Lake Malawi [44], are known as scale eaters. On the other hand, at least nine species of six genera of South American characins are scale-eaters [16], [45]. These African cichlids and South American characins are both apparently polyphyletic. Interestingly, the jaw apparatus of these scale-eaters are diverse even within the same family and imply several trophic origins [16], [43]. Further studies on these cichlid and characin scale-eaters and comparison between them will shed light on the origin of morphological novelties such as jaw asymmetry as well as specialized teeth, and the exploitation of a novel food source.

On the other hand, several fishes have the same morphological laterality working in their asymmetric feeding behavior. In largemouse bass, *Micropterus salmoides*, the righty individuals make counterclockwise attack on the prey fish from the behind, and the lefties do the opposite [8], [27]. In a Tanganyikan cichlid, *Neolamprologus fasciatus*, righty aims at prey shrimp with the right side of the body abutting a rock and darts to the prey rightward, and lefty does the opposite [9]. This may imply that lateral asymmetry in fishes is not limited in scale-eaters, but are more common, and the laterality may have a significant function in individual-based prey-predator interactions. Further research is needed to clarify how this antisymmetry is ubiquitous in fishes, how the antisymmetry in external morphology correlates with behavioral handedness, and whether the morphological laterality of these fishes shares the genetic background.

Asymmetries are ubiquitous in animals, including humans [46], [47], [48]. The asymmetry in animals involves three aspects, that is, asymmetry in the brain and viscera, that in external morphology, and that in behavior. Our knowledge of their linkages remains limited. For example, behavioral handedness is thought to be a result of cerebral asymmetry [48] or a fortuitous consequence of fluctuating asymmetry, that is, the inability to develop symmetrically [49]. In fishes, however, the structural asymmetry in the brain is concordant with the visceral asymmetry in more than 95% [50], showing directional asymmetry, and the laterality is consistent in species [7], [51]. Therefore, this cerebral asymmetry cannot explain behavioral handedness at an individual level in several fishes, such as *Jenynsia lineata* [52], *Betta splendens* [53], and *Gambusia holbrooki* [54], whose populations share the directional cerebral asymmetry but contain both lefty and righty individuals in behavior. Although functional lateralization of the brain can play a role in these fishes, our study indicates that the external antisymmetry in head inclination and mandibles can be a determinant of behavioral handedness. Alternatively it is possible that the behavioral handedness can alter morphological handedness [55]. *Perissodus microlepis* were fed with a bilaterally biased dummy prey, “soft-bait dummy fish wrapped in trout skin and with spikes preventing foraging from the forbidden flank [28]” in an aquarium. However, no obvious result is seen in the difference between before and after the six-month experiment. More study is needed to define whether behavior or morphology determines the other asymmetry, or they interact with each other.

The present study adds to the understanding of animal asymmetry by describing another species of scale eater with laterality that has evolved independently and is comparable to the cichlid system. Further studies of these cichlid and characin scale eaters and comparisons between them will shed light on animal asymmetries in external morphology, behavior, and even cerebral and visceral systems, as well as the linkages among these.

## Supporting Information

Table S1 Scale-eating fishes in freshwater and marine habitats.
(DOC)

Video S1 Scale eating behavior by *Exodon paradoxus.* This individual was righty in jaw morphology and attacked the left flank of the prey gold fish.
(WMV)
