# Supplementary material for: Jaw Laterality and Related Handedness in the Hunting Behavior of a Scale-Eating Characin, Exodon paradoxus
Source: PLoS One. 2011 Dec 28;6(12):e29349. doi: 10.1371/journal.pone.0029349 (PMC3247259; doi:10.1371/journal.pone.0029349)
Supplement: Table S1 — Scale-eating fishes in freshwater and marine habitats. (DOC) [file pone.0029349.s001.doc]

**Table S1**. Scale-eating fishes in freshwater and marine habitats (Sazima 1983; Nakae & Sasaki 2002; Grubh & Winemiller 2004).

Habitat Family Order

Freshwater Characidae Characiformes

Schilbeidae Siluriformes

Ariidae Siluriformes

Trichomycteridae Siluriformes

Cichlidae Perciformes

Marine Ariidae Siluriformes

Ambassidae Perciformes

Blenniidae Perciformes

Teraponidae Perciformes

Labridae Perciformes

Kyphosidae Perciformes

Carangidae Perciformes

Triacanthodidae Tetraodontiformes

References

Grubh, A. & Winemiller, K. (2004) Ontogeny of scale feeding in the Asian Glassfish, *Chanda nama* (Ambassidae). *Copeia* **2004**, 903-907.

Nakae, M. & Sasaki, K. (2002) A scale-eating triacanthodid, *Macrorhamphosodes uradoi*: prey fishes and mouth "handedness" (Tetraodontiformes, Triacanthoidei). *Ichthyol. Res.* **49**, 7-14.

Sazima, I. (1983) Scale-eating in characoids and other fishes. *Environ. Biol. Fish.* **9**, 87-101.
